# Oral health and respiratory disease: A case-control study on periodontal status and quality of life among adults 

**DOI:** 10.6026/973206300212862

**Published:** 2025-08-31

**Authors:** Sonal Subhangi, Pradeep Tangade, Vikas Singh, Ankita Jain, Harshita Pandey, Apeksha Suryawanshi, Rupali Malik

**Affiliations:** 1 Department of Public Health Dentistry, Teerthanker Mahaveer Dental College and Research Centre, Teerthanker Mahaveer University, Delhi Road, Moradabad, Uttar Pradesh, India

**Keywords:** Periodontal health, respiratory health, chronic obstructive pulmonary disease, asthma, pneumonia, bronchitis

## Abstract

Periodontal disease is a chronic inflammatory condition that may contribute to respiratory illnesses such as chronic obstructive pulmonary disease (COPD), asthma, pneumonia and bronchitis. This case-control study of 664 participants assessed the association between periodontal and respiratory health using WHO 2013 criteria for periodontal evaluation, spirometry for respiratory assessment and the Oral Health Impact Profile-14 for quality of life. The mean age of participants was 45.51 years, with males predominating and patients with respiratory disorders showed significantly poorer periodontal status than controls. The worst periodontal health was observed among patients with COPD. A significant correlation between periodontitis and COPD was found, emphasizing the need for risk factor evaluation, patient education and preventive intervention strategies.

## Background:

Respiratory illnesses such as chronic obstructive pulmonary disease (COPD), asthma, pneumonia and bronchitis severely compromise pulmonary function and general quality of life [1, 2- 3]. These conditions are influenced by multiple factors including smoking, environmental toxins, infection and hereditary predisposition [4, 5- 6]. Periodontal disease represents an inflammatory condition affecting oral tissues such as the gingiva, periodontal ligament and alveolar bone [7, 8]. If left untreated, periodontitis can lead to tooth loss and has been implicated in systemic health complications [9, 10]. Emerging evidence indicates a close association between periodontal and respiratory health [11, 12]. Oral pathogens can translocate into the lower respiratory tract through aspiration, contributing to pulmonary infections [13, 14]. Moreover, chronic oral inflammation enhances systemic inflammatory burden, which may exacerbate respiratory conditions [15, 16]. Socioeconomic determinants including income, education and healthcare access further influence the prevalence and severity of both periodontal and respiratory diseases [17, 18-19]. This research investigates the association between respiratory disease and periodontal health in a case-control design. Respiratory function, oral status and quality of life are assessed using the Oral Health Impact Profile-14 instrument. The hypothesis is that patients with respiratory diseases will demonstrate worse periodontal status and lower oral health-related quality of life compared to healthy controls [20, 21-22]. Therefore, it is of interest to describe the association between periodontal disease and respiratory health in adult patients.

## Materials and Methods:

## Study design:

A case-control design was used to investigate the association between periodontal health and respiratory disease. The aim was to compare Oral health-related quality of life and periodontal parameters in patients with diagnosed respiratory diseases and matched controls with no such disease.

## Ethical considerations:

The Institutional Ethical Committee (TMDCRC/IEC/TH/22-23/PHD 01) approved the study and it was carried out based on the Helsinki Declaration (2008). All participants provided informed consent following explanation of the study aims and procedures.

## Study setting:

The research was carried out over a period of one year from 4th May 2023 to 5th May 2024 at Teerthanker Mahaveer Medical Hospital, Moradabad and Uttar Pradesh, India. The participants were recruited from the outpatient department. The research involved processes for initial recruitment, clinical assessment and follow-up and data collection.

## Inclusion criteria:

Participants for the study were people between 16 and 68 years of age. Cases were people who had a medically confirmed diagnosis of a respiratory illness like chronic obstructive pulmonary disease, asthma, pneumonia, or bronchitis. The control group was people who did not have any history or diagnosis of a respiratory illness. Voluntary consent and informed consent from all participants was sought after informing them of the nature and purpose of the study [18].

## Exclusion criteria:

Participation in the study if the participant had systemic diseases that were not related to the research focus. Also excluded were individuals who had taken antibiotics during the preceding weeks before entering the study. Persons who had undergone any periodontal treatment within a period of six months were also excluded to prevent fresh interventions from affecting the results of the study [18].

## Selection of cases and controls:

Cases were chosen by documented medical diagnoses of respiratory disease in order to allow proper classification. Controls were selected by matching them by gender and age in an attempt to avoid confounding. Both were drawn from the same hospital population for the sake of reducing selection bias.

## Matching strategy:

Each case was matched with an age- and gender-matched control within a range of ±2 years. This 1:1 matching approach was used to maximize comparability and minimize confounding between the case and control groups, thus maximizing the internal validity of the study.

## Variables:

The main outcome variables measured in this study were respiratory function, as measured by spirometry; periodontal status, as measured by the World Health Organization criteria [19] and Oral health-related quality of life, as measured by the Oral Health Impact Profile-14 questionnaire [20]. The main exposure variable was the severity of periodontal disease. In order to accurately interpret results, some potential confounders were controlled for, including age, gender, smoking status, socioeconomic status and education level. In addition, the analysis considered potential effect modifiers, including the period and severity of respiratory and periodontal infections that may affect the observed relationships.

## Data collection and measurement:

Periodontal status was measured with World Health Organization (2013) [19] criteria by blinded examiners. Respiratory function was assessed through spirometer by trained technicians on standardized machines. Oral health-related quality of life was recorded with the Oral Health Impact Profile-14 questionnaire in English or Hindi [20].

## Pilot study:

Socio demographic information, such as age, gender, education, residence, socioeconomic status and smoking, was recorded on a structured questionnaire. Prior to the main study, a pilot study was performed on fifty subjects who were found to be eligible. The questionnaires were distributed only to those patients diagnosed with both respiratory disorders (like Chronic Obstructive Pulmonary Diseases, asthma, pneumonia and bronchitis) and periodontal issues, collecting their demographic information and pertinent health information. Pilot study data were not included in the main analysis. Rather, the pilot study was used to improve the scheduling and conduct of the main research, specifically the questionnaire-based case-control survey. The results of the pilot study were carefully examined and necessary adjustments were done to the questionnaire according to the findings. The internal consistency of the questionnaire was tested using Cronbach's alpha, which gave a value of α = 0.80, reflecting good internal consistency of the instrument.

## Bias control:

To reduce selection bias, controls were meticulously matched to cases drawn from the same hospital population and therefore were comparable between the groups. Measurement bias was minimized through the employment of standardized protocols and instruments, combined with adequate training of examiners to ensure consistency of data collection. Observer bias was avoided with the use of calibration sessions and the adoption of standardized data collection procedures, promoting reliability and consistency among all the assessments.

## Sample size calculation:

Sample size was estimated using the formula-

N = (Z^2^PQ) / d^2^

Where, Z is the standard normal deviate for a 95% confidence level (Z = 1.96), P is the pilot data estimated prevalence (25%), Q is the remainder of P (100 - 25 = 75) and d is the acceptable error margin (8%). The minimum number of samples per group was 112. For a 20% dropout, the target sample was raised to 140 per group. In the end, 332 were enrolled in each group, a total of 664.

## Participant flow and study attrition:

The research started with 1,120 potentially eligible participants, out of which 1,008 were screened for eligibility excluding those with incomplete information, non-response, or contrary treatments. After checking for eligibility, 907 participants were verified as eligible, 101 being excluded for ineligibility on medical grounds or refusal to have required examinations. Among the eligible participants, 816 participants consented to participate in the study and 91 did not because of time constraints, fear of procedures, or privacy. At the follow-up stage, 734 participants finished the study and 82 dropped out because of relocation, worsening of health, or loss of interest. Ultimately, 664 participants were analysed.

## Handling of quantitative variables:

Quantitative measures like gingival index, probing depth, attachment loss and spirometer values were categorized according to clinical cut-offs for easier interpretation. Comparisons within groups were made between cases and controls as well as between disease subgroups.

## Statistical analysis:

All statistical comparisons were carried out using SPSS software version 16.0. Depending on the data type, parametric and non-parametric tests were used. Parametric tests involved one-way ANOVA and the independent t-test, whereas Kruskal-Wallis and Mann-Whitney U tests were used for non-parametric data. To control for possible confounders, multivariate logistic regression analysis was used. Missing values were handled with suitable imputation techniques to maintain the integrity of the data. A p-value below 0.05000 was used as statistically significant. Moreover, sensitivity analyses were conducted to evaluate the reliability and robustness of the findings.

## Results:

The survey consisted of 664 participants between 16 and 68 years old. Out of these, there were 400 (60%) males and 264 (40%) females, giving a suitable gender-based sample for analysis. The socioeconomic background of the 664 participants shows a distribution towards the lower to middle strata, with 22% lower class, 27% lower-middle class and 21% upper-lower class. The upper-middle and upper classes together made up 18% and 12% of the participants, respectively. As far as residence goes, most 56% came from urban locations, with 44% having rural backgrounds, a difference which usually accompanies disparate access to healthcare, education and infrastructure. As far as educational attainment goes, the group of participants exhibited a fairly high degree of formal education: 24% were 10th-grade graduates, 26% 12th-grade graduates, 43% graduates and 7% postgraduates. Lastly, the intent of hospital visits reflected a compromise between follow-up care (58%) and treatment visits (42%), indicating preventive and therapeutic healthcare involvement among the participants. On practices of oral hygiene, the employ of different types of aids were noticed among participants. Although most prevalent were the toothbrushes (53%), age-old approaches such as usage of a finger (25%) or neem stick/tree twig (22%) were also utilized, reflecting an amalgamation of contemporary and customary practices. In analysing the cleaning materials used, toothpaste was used most often (44%), yet common materials like charcoal (30%), toothpowder (18%) and salt (9%) still played a role. The rate of brushing showed that most (56%) brushed once daily, with a high percentage (44%) brushing twice daily, which indicates an overall good frequency of oral hygiene. As far as brushing technique was concerned, vertical strokes were used by 42% of the subjects, horizontal strokes by 35% and a combination technique by 24%, with vertical and horizontal techniques being the predominant methods. The results of this case-control study offer strong evidence of a relationship between socio demographic variables, oral hygiene behaviours and systemic health outcomes, specifically for respiratory health. Table 1 (see PDF) shows a strong correlation between respiratory illness and periodontal condition. Applying the Kruskal-Wallis test, all clinical indices-gingival index, probing depth and attachment loss-were strongly significant (p < 0.01) with the highest values in the patients with Chronic Obstructive Pulmonary Diseases. Table 2 (see PDF) gives the correlation between oral health-related quality of life and respiratory diseases. While the highest scores on OHIP-14 were noted in Chronic Obstructive Pulmonary Diseases patients, they were not statistically significantly different among the groups (p > 0.05) according to one-way ANOVA. Table 3 (see PDF) summarizes periodontal status and Oral health-related quality of life in patients with and without respiratory disease. Independent t-tests revealed considerably higher gingival index, pocket depth and loss of attachment among respiratory patients (p < 0.01), whereas OHIP-14 scores were statistically comparable across the two groups (p > 0.05), revealing that respiratory disease did not have significant effects on subjective oral health quality of life.

## Discussion:

The key findings of this study indicate that there is a strong association between periodontal disease and respiratory disease, especially Chronic Obstructive Pulmonary Diseases (COPD). Participants with respiratory diseases had poorer periodontal health as indicated by higher gingival index scores, increased probing depth and greater attachment loss. Although mean oral health-related quality of life scores were highest among COPD patients, case-control differences were not statistically significant. These findings suggest that periodontal status could be an etiologic factor in the pathophysiology or exacerbation of respiratory diseases and periodontal therapy may improve respiratory outcomes. Our results are consistent with previous research by Scannapieco *et al.* [21, 22], who hypothesized that oral bacteria cause respiratory infections through aspiration and systemic inflammation. Similarly, Sharma *et al.* [23], Wang *et al.* [24] and Kowalski *et al.* [25] reported that patients with COPD and other respiratory diseases had worse periodontal status than healthy subjects. The increased inflammatory load in periodontitis could contribute to airway infection and inflammation, particularly in vulnerable groups such as the elderly, smokers and those with poor access to dental care. Supporting this, Watando *et al.* [26] demonstrated that improved oral hygiene habits significantly reduced the prevalence of pneumonia in elderly nursing home residents, affirming the protective role of periodontal treatment in preventing systemic disease. Biologically, the relationship between periodontal and respiratory illnesses can be explained by several mechanisms. First, aspiration of oral pathogens into the lower respiratory tract directly introduces bacteria that may trigger respiratory infections. Second, systemic inflammation mediated by cytokines in periodontal disease may increase airway reactivity and exacerbate respiratory illness. Finally, periodontal disease may facilitate breakdown of mucosal barriers in the respiratory tract, allowing easier colonization by respiratory pathogens. These mechanisms are substantiated by evidence from clinical and experimental studies, as shown by Hujoel *et al.* [27] and Seymour *et al.* [6]. Although this study is significant, several limitations must be considered. The cross-sectional design prevents determination of causality or the direction of association between respiratory and periodontal diseases. While the sample size was sufficient, broader and more diverse populations would increase generalizability. The short study duration may not fully capture the chronicity of disease progression. Reliance on self-reported smoking and oral hygiene practices also risks recall bias. Furthermore, unmeasured confounders such as stress, immune status, or diet may have influenced results. Socioeconomic disparities in dental care and the impact of smoking, despite statistical control, remain important determinants. While the study involved a large, demographically diverse cohort that strengthens internal validity, its single tertiary care centre design limits external validity. Future research should involve multi-centric, longitudinal cohorts to confirm these findings and establish causality. Larger, more representative populations are required, along with randomized controlled trials to examine the effect of periodontal therapy on respiratory outcomes and studies in non-smokers to clarify its independent role in systemic inflammation. The present results highlight the importance of integrating oral health evaluation into respiratory care guidelines. Public health initiatives must emphasize oral hygiene as a modifiable risk factor in preventing respiratory disorders. Clinicians should prioritize screening at-risk patients for periodontal infection, include dental professionals in interdisciplinary care teams and educate patients on the oral-systemic health connection.

## Conclusion:

An important connection between periodontal disease and respiratory illness, including Chronic Obstructive Pulmonary Diseases and highlights the influence of oral health on systemic health. Thus, we show incorporating periodontal screening into respiratory care guidelines. Investing in oral health is not merely about smiles it's about easier breathing and better living. Additional research is needed to determine the potential for periodontal therapy to enhance respiratory outcomes.

## Funding:

NIL
